# A Dual-Modality Home-Based Cardiac Rehabilitation Program for Adults With Cardiovascular Disease: Single-Arm Remote Clinical Trial

**DOI:** 10.2196/59098

**Published:** 2024-10-01

**Authors:** Tim Bilbrey, Jenny Martin, Wen Zhou, Changhao Bai, Nitin Vaswani, Rishab Shah, Sara Chokshi, Xi Chen, Satjit Bhusri, Samantha Niemi, Hongdao Meng, Zhen Lei

**Affiliations:** 1 RecoveryPlus.Health, Inc New York, NY United States; 2 Node.Health Foundation Wilmington, DE United States; 3 Upper East Side Cardiology PLLC New York, NY United States; 4 McCormick School of Engineering Northwestern University Chicago, IL United States; 5 School of Aging Studies University of South Florida Tampa, FL United States

**Keywords:** cardiac rehabilitation, telehealth, mHealth, digital health, exercise, quality of life, myocardial infarction, app, application, physical fitness, cardiac rehabilitation, self-management, disease management

## Abstract

**Background:**

Cardiac rehabilitation (CR) is a safe, effective intervention for individuals with cardiovascular disease (CVD). However, a majority of eligible patients do not complete CR. Growing evidence suggests that home-based cardiac rehabilitation (HBCR) programs are comparable in effectiveness and safety with traditional center-based programs. More research is needed to explore different ways to deliver HBCR programs to patients with CVD.

**Objective:**

We aimed to assess the feasibility and impact of a digital HBCR program (RecoveryPlus.Health) that integrates both telehealth and mHealth modalities on functional exercise capacity, resting heart rate, and quality of life among adults with CVD.

**Methods:**

This 12-week prospective, single-arm remote clinical trial used a within-subject design. We recruited adults with CVD (aged ≥40 years) from the community with a CR-eligible diagnosis (stable angina pectoris, myocardial infarction, and heart failure) between May and August 2023. All enrolled patients referred to the RPH clinic in Roanoke, Texas, were included. The care team provided guideline-concordant CR services to study participants via two modalities: (1) a synchronous telehealth exercise training through videoconferencing; and (2) an asynchronous mobile health (mHealth) coaching app (RPH app). Baseline intake survey, electronic health record, and app log data were used to extract individual characteristics, care processes, and platform engagement data. Feasibility was measured by program completion rate and CR service use. Efficacy was measured by changes in the 6-minute walk test, resting heart rate, and quality of life (12-Item Short-Form Health Survey) before and after the 12-week program. Paired *t* tests were used to examine pre- and postintervention changes in the outcome variables.

**Results:**

In total, 162 met the inclusion criteria and 75 (46.3%) consented and were enrolled (mean age 64, SD 10.30 years; male: n=37, 49%; White: n=46, 61%). Heart failure was the most common diagnosis (37/75, 49%). In total, 62/75 (83%) participants completed the 12-week study and used the telehealth modality with 9.63 (SD 3.33) sessions completed, and 59/75 (79%) used the mHealth modality with 10.97 (SD 11.70) sessions completed. Post intervention, 50/62 (81%) participants’ performance in the 6-minute walk test had improved, with an average improvement of 40 (SD 63.39) m (95% CI 25.6-57.1). The average 12-Item Short-Form Health Survey’s physical and mental summary scores improved by 2.7 (SD 6.47) points (95% CI 1.1-4.3) and 2.2 (SD 9.09) points (95% CI 0.1-4.5), respectively. There were no changes in resting heart rate and no exercise-related adverse events were reported.

**Conclusions:**

The RecoveryPlus.Health digital HBCR program showed feasibility and efficacy in a group of nationally recruited patients with CVD. The findings add to the evidence that a telehealth and mHealth dual-modality HBCR program may be a promising approach to overcome some of the main barriers to improving CR access in the United States.

**Trial Registration:**

ClinicalTrials.gov NCT05804500; https://clinicaltrials.gov/search?cond=NCT05804500

## Introduction

Cardiovascular disease (CVD) remains the leading cause of morbidity and mortality in the United States, with more than 2 million hospitalizations and 400,000 deaths due to CVD [1,2]. Cardiac rehabilitation (CR) is a grade I guideline-recommended multidisciplinary program intended to reduce the risk of subsequent cardiovascular events and improve quality of life through activities shown to improve physical, psychological, and social functioning, and ultimately reduce associated morbidity among patients with CVD [3,4]. Despite strong evidence regarding its safety and efficacy, CR use rate remains very low, with only about 1 in 4 eligible patients enrolling in CR [5-10]. In addition, disparities in CR uptake with respect to sex, age, race, ethnicity, and geographic locations persist, contributing to the low overall CR participation rate and substantial geographic variations in participation [3,11]. With the initiation of the Million Hearts 2022, a national program aimed at achieving a target of 70% CR participation among eligible patients by 2027 [9], the current 2300 center-based cardiac rehabilitation (CBCR) programs in the United States are unlikely to meet the demand for CR [12,13]. Therefore, new home-based cardiac rehabilitation (HBCR), an alternative to traditional CBCR that allows patients to undergo rehabilitation in their own homes, is urgently needed to expand CR access [3,14].

HBCR alone or in combination (hybrid) with CBCR, has been shown to be a safe and effective option for patients eligible for CR [15-17], with the potential to address some of the key barriers to CR access (eg, time and cost of transportation) [5]. HBCR programs use the same multifaceted approaches (exercise training, education, dietary guidance, and lifestyle modification) as CBCR, providing patients with synchronous or asynchronous delivery of supervised remote exercise training and care management [4,18,19].

While HBCR programs have certain disadvantages compared with CBCR (eg, lack of published clinical standards, lower exercise training intensity, and potential safety concerns for high-risk patients), their key advantages include reduced enrollment delays, expanded capacity, flexible scheduling, minimal travel, and improved access for patients with limited mobility [2,5,20]. The unexpected disruption to CBCR access caused by the COVID-19 pandemic inadvertently accelerated the transition of many in-person CR programs to the home setting and increased the acceptability of HBCR and center-home hybrid CR programs [21]. There is a need for greater engagement in safe and evidence-based CR exercise programming for patients recovering from CVD. Among these new approaches, home-based synchronous remote CR and asynchronous mobile health (mHealth)–based CR modalities, alone or in combination, have the potential to improve access by expanding provider offerings and reduce the time and costs associated with travel to traditional CR centers [22].

The RecoveryPlus.Health digital (RPH-D) HBCR program is an innovative program that combines 2 delivery modalities, a synchronous telehealth modality delivered through videoconferencing by an interdisciplinary care team and an asynchronous mHealth modality delivered through a digital app (RPH app) for individualized exercise therapy. The RPH-D program was developed based on the American Heart Association CR guidelines for delivering tailored, evidence-based CR remotely and on demand.

This study aimed to determine whether the dual-modality RPH-D program is feasible for delivering remote CR to a nationally recruited sample, and to examine its impact on patients’ functional exercise capacity (cardiorespiratory endurance), resting heart rate, and quality of life.

## Methods

### Recruitment

This is a prospective single-arm remote clinical trial using a within-subject design. Between May 1, 2023, and November 30, 2023, a dedicated recruitment website was established to facilitate the sharing of study information for recruitment through 2 primary channels, referrals from cardiology providers in the Dallas-Fort Worth area and through a collaboration with The Mended Hearts, Inc, a national and community-based nonprofit cardiovascular patient support organization. The community partners sent emails to their patients prompting potential participants to visit the study website. Potential participants were screened for eligibility criteria by a secure web-based questionnaire. Patients who passed the initial screening were contacted by the study coordinator by phone to verify eligibility and finalize enrollment.

Participants were included if they were aged 45 years or older; had stable CVD and under medical management; received referral to CR from a provider within 60 days; were able to walk unassisted; deemed stable with low to moderate risk of a cardiac event; and with a CR eligible diagnosis in previous 12 months as defined by Medicare Part B (stable angina pectoris, myocardial infarction, stable heart failure, and coronary artery bypass graft surgery). Participants were excluded if they were unable to read and speak in English, had a BMI greater than 40 kg/m^2^, had hospitalization or significant decline in health, had physical or mental health limitations that prohibit participation in exercise activities, could not use a tablet computer, lack of access to Wi-Fi, or had severe hearing or vision impairment.

### Intervention: The RecoveryPlus.Health Digital CR Program

The RPH-D program for patients recovering from a CVD event leverages 2 weekly concurrent remote modalities to engage the study participants: (1) synchronous (telehealth) coaching sessions conducted by an exercise physiologist by Zoom for Healthcare (Zoom Video Communications, Inc); and (2) asynchronous (mHealth) exercise sessions by the RPH app. The telehealth sessions focused on the following core CR services: patient assessment, exercise training, care management, lifestyle counseling, and remote monitoring. The study iPads were preloaded with the RPH app, which offers a library of on-demand exercise videos with varying degrees of difficulty. Wireless heart rate and blood pressure monitors were connected to the iPad by Bluetooth to enable real-time monitoring of patient vital signs and feedback (eg, patient-rated difficulty for each prescribed exercise) and tailor individual patient regimens regularly for safety and fine-tuning the exercise therapy. During the app-based exercise sessions, participants rated the perceived level of difficulty (exertion) of each exercise on a 1-10 scale. If a patient chooses to skip an exercise or exit a session before completing the prescribed exercises, the reasons were documented by the app (Multimedia Appendix 1).

All clinical team members had access to the RPH remote health care provider web portal (HIPAA [Health Insurance Portability and Accountability Act]-complaint) to enter, store, and view participant demographic and clinical information, create fitness assessments, write and update a patient’s exercise prescription, and generate reports. The clinicians were trained to use patient feedback along with data derived from heart rate monitors to assess exercise intensity, appropriateness, and adherence to the prescribed exercise program, and, importantly, make adjustments to better personalize the care plan for each patient. The RPH remote CR platform also provided real-time, automated alerts that were sent to the care team for any symptoms or reports of out-of-range heart rate reading to facilitate immediate evaluation to determine the severity of a symptom and which course of action is required for follow-up. In these events, participants were instructed to stop exercising immediately and report any cardiac-related symptoms to their clinician at any time, during or outside of exercise sessions. The clinical care team received and responded to alerts 24 hours a day, 7 days per week.

### Outcomes and Measures

The main variables of interest were feasibility and efficacy. Feasibility was measured by program initiation (the proportion of enrolled patients who completed all baseline assessments and completed the initial Zoom teleconferencing session with an exercise physiologist and participation (the proportion of enrolled patients who completed at least 50% or more of prescribed sessions). Feasibility data were captured by the HIPAA-compliant, encrypted, RPH electronic health records. Efficacy was measured by 6-minute walk test (6MWT), resting heart rate, and quality of life. These measures were assessed by the study exercise physiologist and recorded on the RPH electronic health record platform. The 6MWT was measured by the 6WT app, an iOS app validated in 330 volunteers (age range 16-91 years) [23]. Participants were instructed to walk on a flat, hard surface outside of their homes for a period of 6 minutes. The 6MWT is a widely used and well-documented measure of aerobic exercise capacity used to indicate change in fitness [24]. Resting heart rate is positively associated with mortality and is known to decline with regular exercise [25]. Quality of life was measured by the 12-Item Short-Form Health Survey (SF-12, version 2), a validated and commonly used measure of perceived quality of life and functional health among patients with CVD [26]. Individual characteristics included basic sociodemographic variables collected at baseline (age, sex, race, ethnicity, comorbidities, and referring diagnosis).

### Sample Size Calculation

Power calculation was based on the primary efficacy measure: 6MWT. At a 5% level of significance, to achieve 85% power in detecting a small to moderate difference of 35 m for the 6MWT before and after the RPH-D program, a sample size of 75 participants was required [24,27]. To account for an estimated 25% attrition rate, a total sample of 100 was set as the enrollment target.

### Statistical Analysis

Standard descriptive statistics were used to describe the study sample and compare them by study completion status. For continuous variables, 2-sample *t* test was used, and Fisher exact test was used for categorical variables. To examine the impact of the RPH-D program on the outcome measures, paired *t* tests were used to examine whether there were any within-subject changes post intervention. To quantify the uncertainty surrounding the point estimates of effect sizes, 95% CI was calculated using nonparametric bootstrapping with 5000 repetitions [28]. Statistical analysis was performed in Stata 17 BE (StataCorp).

### Ethical Considerations

The study protocol was approved by the Advarra Institutional Review Board (Pro00070335) and written informed consent was obtained from all participants.

## Results

### Overview

**Figure 1:**
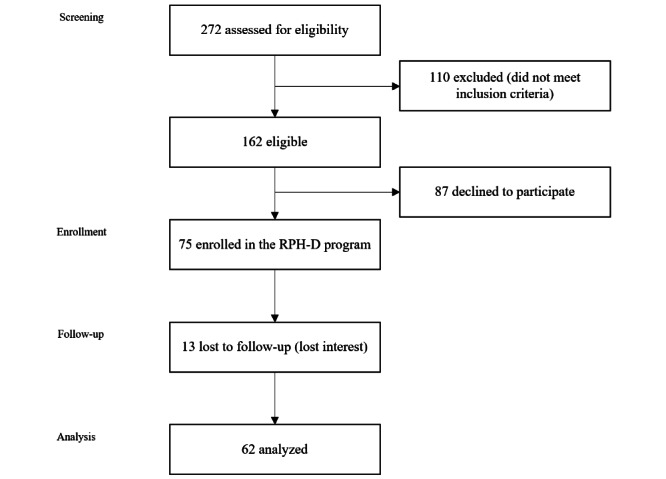
CONSORT (Consolidated Standards of Reporting Trials) flow diagram for patient enrollment and follow-up. RPH-D: RecoveryPlus.Health Digital.

Of the 272 individuals screened for eligibility, a total of 162 individuals from 29 states met the study eligibility criteria and 75 (46%) consented to the study and were enrolled in the RPH-D program. In total, 13 participants withdrew from the study and 62 (83%) were included in the analysis (Figure 1).

### Participant Characteristics

Table 1 displays the baseline participants’ individual characteristics for the overall sample and by participation rate (less than or greater than 50%). Study participants had a mean age of 64.6 (SD 10.0; range: 45-85 years) and 38 (51%) were female (Table 1). Non-Hispanic White participants made up majority of the sample at 46 (61%), followed by 8 (11%) Black American, 5 (7%) Hispanic, and 16 (21%) other or missing race. The top 5 referring diagnoses were chronic heart failure (n=37, 50%), coronary artery bypass grafting (n=9, 15%), valvular surgery (n=9, 15%), acute myocardial infarction (n=6, 10%), and percutaneous coronary intervention (n=6, 10%). Participants who completed 50% or more sessions were older and more likely to have percutaneous coronary intervention as the referring diagnosis. With the exception of age and BMI, there were no significant differences in the characteristics of the participants who completed the study versus those who did not.

A total of 62 out of 75 (83%) participants completed the 12-week study. Out of 62 participants, 50 (81%) completed at least 50% of CR sessions. All participants were prescribed 12 one-on-one videoconferencing (telehealth) sessions (55 minutes per week, 660 minutes total) and 12 app-based exercise (mHealth) sessions (27.8 minutes per week, 334 minutes total) over 12 weeks. A total of 62 out of 75 (83%) participants used the telehealth modality (9.63, SD 3.33 sessions) and 34 of 62 (55%) participants completed all 12 sessions. A total of 59 out of 75 (79%) used the mHealth modality (10.97, SD 11.70 sessions) and 32 out of 62 (52%) completed all 12 sessions. Among those who completed the study, all participants used the telehealth modality and all but 3 participants used the mHealth modality. Participants completed an average of 654.1 (SD 113.6) minutes of telehealth sessions and 421 minutes (SD 306) of mHealth sessions (Table 2). A total of 12 participants visited the emergency room (15 visits total) and none were related to the RPH-D program.

In total, 3 outcome measures were used to examine the impact of the intervention before and after the 12-week intervention (Table 3). Overall, participants who completed the intervention had an average improvement in 6MWT performance of 40 (SD 63.39) meters (95% CI 25.6-57.1). The average SF-12 physical and mental component summary scores improved by 2.7 (SD 6.47, 95% CI 1.1-4.3) and 2.2 (SD 9.09, 95% CI 0.1-4.5) points, respectively. There was a small nonsignificant improvement in average resting heart rates (mean –1.1, SD 9.05, 95% CI –3.4 to 1.1).

**Table 1:** Baseline characteristics of study participants (N=62).

Variables		Overall (N=62)	<50% completion (n=12)	≥50% completion (n=50)	*P* value
Age (years), mean (SD)		64.6 (10)	56.9 (6.1)	66.4 (9.9)	<.001
**Age group (years), n (%)**					
	45-54	12 (19.4)	4 (33.3)	8 (16)	—^a^
	55-64	20 (32.3)	7 (58.3)	13 (26)	—^a^
	65-74	19 (30.6)	1 (8.3)	18 (36)	—^a^
	75-85	11 (17.7)	0 (0)	11 (22)	—^a^
**Sex, n (%)**					.75
	Male	30 (48.4)	5 (41.7)	25 (50)	—^a^
	Female	32 (51.6)	7 (58.3)	25 (50)	—^a^
**Race/ethnicity, n (%)**					
	Non-Hispanic White	37 (59.7)	8 (66.7)	29 (58)	—^a^
	Non-Hispanic Black	7 (11.3)	2 (16.7)	5 (10)	—^a^
	Hispanic	5 (8.1)	1 (8.3)	4 (8)	—^a^
	Other/missing	13 (21)	1 (8.3)	12 (24)	—^a^
BMI (kg/m^2^), mean (SD)		29.3 (6.8)	30.4 (8.2)	29 (6.5)	<.001
**BMI group (kg/m^2^), n (%)**					
	Normal weight	19 (31.1)	5 (41.7)	14 (28.6)	—^a^
	Overweight	19 (31.1)	2 (16.7)	17 (34.7)	—^a^
	Obese	23 (37.7)	5 (41.7)	18 (36.7)	—^a^
**Referring diagnosis, n (%)**					
	Coronary artery bypass grafting	9 (14.5)	2 (16.7)	7 (14)	>.99
	Chronic heart failure	31 (50)	5 (41.7)	26 (52)	.75
	Acute myocardial infarction	6 (9.7)	3 (25)	3 (6)	.08
	Valvular surgery	9 (14.5)	2 (16.7)	7 (14)	>.99
	Angina pectoris	1 (1.6)	0 (0)	1 (2)	>.99
	Percutaneous coronary intervention	6 (9.7)	0 (0)	6 (12)	.59

^a^—: not applicable.

**Table 2:** Participant engagement with the 2 modalities of remote cardiac rehabilitation (N=62).

Variables		Telehealth modality	Mobile health modality
**Number of users, n (%)**			
	Nonusers	0 (0)	3 (4.8)
	Users	62 (100)	60 (95.2)
**Completed number of sessions**			
	Mean (SD)	10.9 (2.3)	12.9 (11.9)
	Median (IQR)	12.0 (10-12)	12 (2-19)
	Range	4-12	0.0-46.0
**Completed total number of minutes**			
	Mean (SD)	654.1 (113.6)	421 (306)
	Median (IQR)	712 (600-720)	391.5 (121.0-636.0)
	Range	219-780	24-1118

**Table 3:** Outcome measures at baseline, 12 weeks, and changes (N=62).

Variables	Baseline	12 weeks	Change, mean (95% CI)
6MWT^a^, meters, mean (SD)	422.8 (78.6)	462.8 (77.4)	40.0 (25.6 to 57.1)
Resting heart rate, beats per minute, mean (SD)	70.6 (12.2)	69.5 (10.6)	–1.1 (–3.4 to 1.1)
SF-12^b^ physical component summary, points, mean (SD)	41.5 (9)	44.2 (8.9)	2.7 (1.1 to 4.3)
SF-12^b^ mental component summary, points, mean (SD)	51.2 (10.8)	53.4 (9.5)	2.2 (0.1 to 4.5)

^a^6MWT: 6-minute walk test.

^b^SF-12: 12-Item Short-Form Health Survey.

## Discussion

### Principal Findings

In this remote clinical study, the RPH digital home-based CR program that integrated synchronous telehealth and asynchronous mHealth modalities was tested among a group of nationally recruited individuals with cardiovascular disease. Screening, recruitment, and engagement outcomes showed that while enrollment yield was modest, the great majority of consented individuals were able to complete the program. Adherence to both modalities was high, although there were substantial variations in adherence to the mHealth exercise sessions. Overall, the program was feasible and efficacious in improving functional exercise capacity and quality of life.

Home-based versus center-based cardiac rehabilitation continues to receive much attention, with a recent Cochrane review identifying 24 completed trials and at least 14 more registered. While the extent of evidence suggests that these 2 delivery approaches showed comparable effects on total morality and exercise capacity up to 12 months postintervention, with no significant differences in health-related quality of life for up to 24 months [12], evidence regarding the delivery modality for HBCR is less clear. A recent review of asynchronous and synchronous delivery models for HBCR showed that while most studies of HBCR tested asynchronous approaches before 2016, a growing number of HBCR trials started to incorporate synchronous approaches [14]. The findings of this study add to the growing evidence base supporting the feasibility of a concurrent dual-modality remote CR for individuals with CVD. For example, a study in New Zealand among predominantly male patients with CHD found that 82.9% (68/82) of those assigned to the remote CR program completed the 12-week program. A total of 2 small studies in the United States reported varying completion rates. Misra et al [29] tested a digitally delivered remote CR program among 12 patients with atrial fibrillation who underwent catheter ablation in Charlotte, North Carolina. In total, 92% (11/12) were able to complete the 12-week program, with an average of 2.9 exercise sessions per week. Giggins et al [30] reported a completion rate of 72.7% (8/11) for an 8-week web-based remote CR program among patients with CHD. Taken together, these findings support the feasibility of HBCR programs for individuals with CVD.

The broad geographic reach (29 states) of the RPH-D program conducted from a single clinic shows the promise of digital technology-enabled remote CR programs. As geographic disparities in access to traditional center-based CR have been highlighted as one of the main barriers to CR participation [3,31], asynchronous home-based CR programs like the RPH-D program provide greater flexibility for patients with CVD. The findings indicate that the great majority of eligible participants-initiated CR with high completion rates. The availability of the asynchronous mHealth modality may help in narrowing the digital divide among patients from rural and remote areas [32]. Participation rate (of 50%) did not differ among participants based on sex and race, suggesting that the program may offer women and minority patients an equitable option for HBCR access.

The findings from this study are also in line with those from multiple systematic reviews concluding that eHealth delivery of cardiac rehabilitation increases patient physical activity and compliance [14,33-35]. As suggested by a recent review of remote solutions for CR, the use of multiple devices for monitoring and communication with their health care team, program personalization, and continuous feedback for users all contributed to the program compliance and feasibility found in this study [20]. This review of 19 different studies of remote cardiac rehabilitation programs noted, as did ours, no intervention-related adverse events, suggesting that remote CR is generally safe.

This study contributes to the literature in several ways. To the best of our knowledge, this is the first study that tested a concurrent telehealth and mHealth dual-modality HBCR program in a group of age and sex-diverse adults in the United States. An Australian-based randomized controlled trial with 24-week (12+12) sequential dual modality HBCR program is currently being tested [36]. It was designed to be pragmatic, with care team members providing remote CR service using existing clinical procedures and electronic medical records system for workflow management. Hybrid, remote, and digitally supported CR services appear to be a safe and effective delivery approach for secondary prevention of CVD with growing evidence [4,37-40]. In addition, the encouraging uptake of remote CR services by women and minority patients may offer new ways to reduce disparities in CR care [41], with the potential to greatly increase the number of patients with CVD engaging in evidence-based CR, an intervention shown to positively impact the health, fitness, and quality of life outcomes for these patients [7,42,43]. Our data demonstrate that patients with CVD will maintain engagement with a platform like RPH remote CR that combines synchronous 1:1 telerehabilitation sessions with an EP with asynchronous exercise sessions longer than they would be traditional in-person CR. In addition, the on-demand exercise feature of the program provides an engaging physical activity option outside of the one-on-one teleconferencing sessions, a promising solution to scheduling and scalability challenges of traditional in-person CR programs. Finally, it is encouraging that this study enrolled a gender, culturally, and geographically diverse patient population, all of which has long been recognized as being underserved by CR services [44-46].

### Limitations

Several limitations should be considered when interpreting the findings. This single-arm remote clinical trial did not include a control group, so we cannot rule out the possibility that the changes in the patient outcome measures may be due to factors other than the RPH-D program. This design choice was based on clinical and ethical reasons: CR is a well-established clinical approach to manage CVD; thus, a randomized controlled trial would have required either not offering or delaying the active intervention to half of all eligible patients. Even a randomized controlled trial with an active CBCR arm would have required some patients to access center-based CR, which may not be readily available to this patient population. Second, study participants came from a national convenience sample with heterogeneous CR-eligible diagnoses and the sample size was modest. Therefore, the findings should be replicated in larger national studies that would allow for subgroup analyses (eg, by referring diagnoses, age, or sex) to help inform clinical practice. Third, the 12-week follow-up period was limited and cannot offer evidence regarding longer-term adherence and outcomes. In addition, the participation rate for the mHealth modality was limited, as some participants experienced challenges with using the iPad with wireless monitors due to limited digital literacy. Finally, relying on 2 primary recruitment channels (through community partner referral and web-based advertisement) contributed to the under-recruitment of the study. Future studies should broaden the referral network to include primary and specialty care and include an economic evaluation component to generate evidence regarding the value for payers [47]. Devices that enable coverage beyond traditional Wi-Fi technology (such as those based on 5G mobile phone network or satellite network) can further reduce geographic barriers to HBCR. These limitations notwithstanding, this is the first US study to test an innovative dual-modality remote CR program that combined synchronous and asynchronous approaches to deliver a fully remote home-based CR program.

### Conclusion

The results of this study show that a dual-modality home-based CR program can be a feasible option to improve access to home-based CR for improving functional exercise capacity after acute CVD. Future research using randomized controlled design (eg, a preference-based RCT) is needed to test the RPH-D CR program in a larger, more diverse pool of patients. If the long-term impact of this program can be confirmed in a multicenter randomized controlled study, a stronger case can be made to implement HBCR programs in health care systems to increase the uptake of CR and bridge the gap between evidence and practice in secondary prevention of CVD.

## Appendix

Multimedia Appendix 1 RecoveryPlus.Health App screenshots.

## Abbreviations

6MWT: 6-minute walk test
CBCR: center-based cardiac rehabilitation
CR: cardiac rehabilitation
CVD: cardiovascular disease
HBCR: home-based cardiac rehabilitation
HIPAA: Health Insurance Portability and Accountability Act
mHealth: mobile health
RPH-D: RecoveryPlus.Health Digital
SF-12: 12-Item Short-Form Health Survey
