# Supplementary figures and images for: A Dual-Modality Home-Based Cardiac Rehabilitation Program for Adults With Cardiovascular Disease: Single-Arm Remote Clinical Trial
Source: JMIR Mhealth Uhealth. 2024 Oct 1;12:e59098. doi: 10.2196/59098 (PMC11480683; doi:10.2196/59098)

Multimedia Appendix 1. RecoveryPlus.Health App screenshots


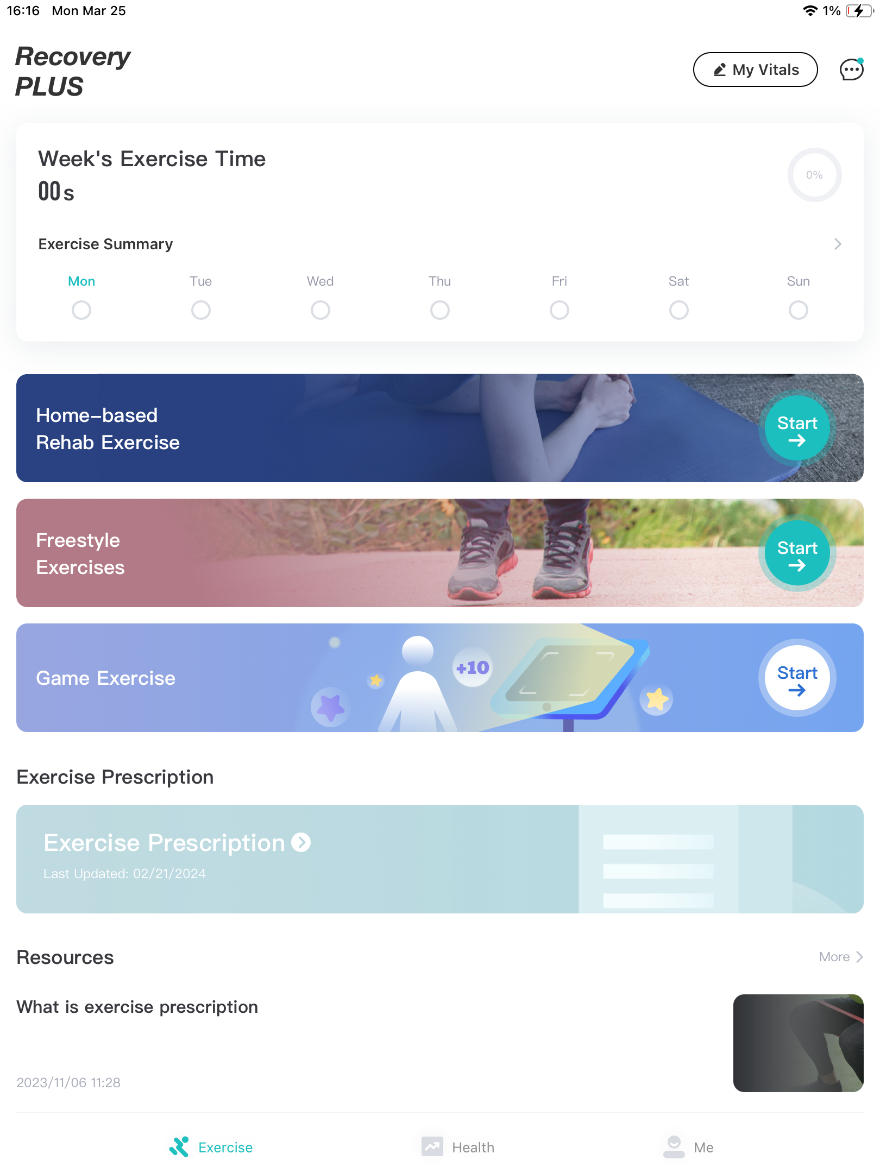


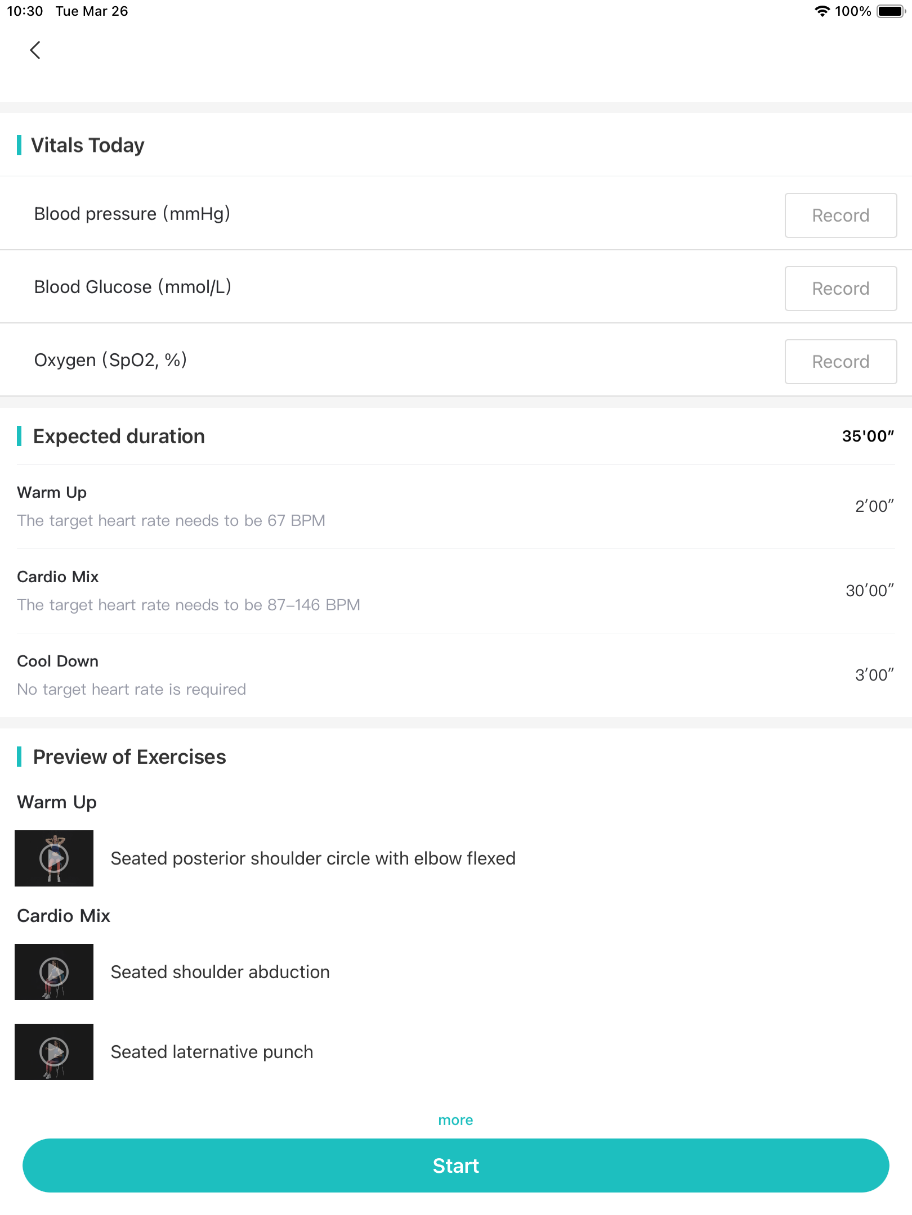


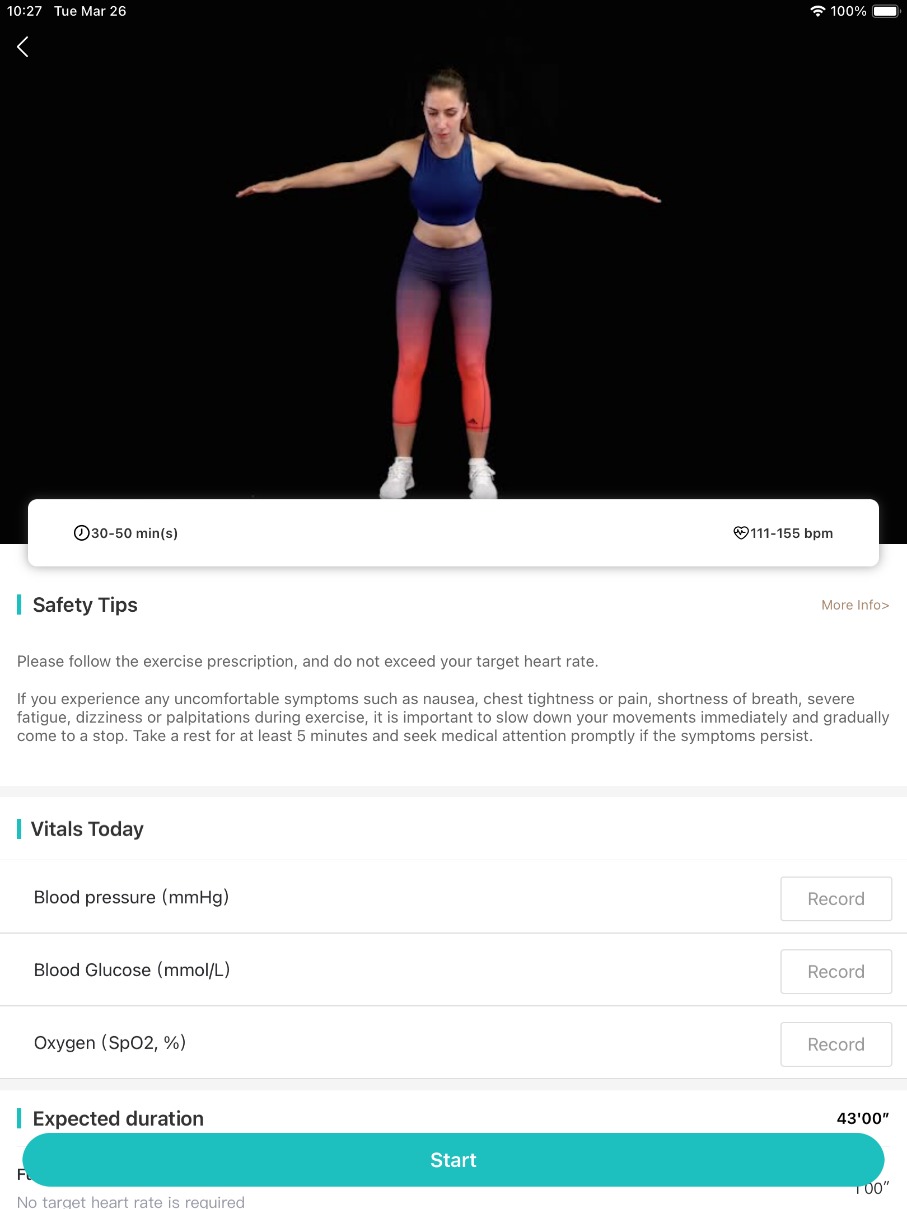

Supplement: Multimedia Appendix 1 [file mhealth_v12i1e59098_app1.docx]
